# Biodistribution of mesenchymal stromal cell-derived extracellular vesicles administered during acute lung injury

**DOI:** 10.1186/s13287-023-03472-8

**Published:** 2023-09-13

**Authors:** Alvin Tieu, Duncan J. Stewart, Damian Chwastek, Casey Lansdell, Dylan Burger, Manoj M. Lalu

**Affiliations:** 1 Department of Cellular and Molecular Medicine, University of Ottawa, Ottawa, ON K1H 8L1 Canada; 2 Department of Anesthesiology and Pain Medicine, University of Ottawa, Ottawa, ON K1H 8L1 Canada; 3 Department of Medicine, University of Ottawa, Ottawa, ON K1H 8L1 Canada; 4 Clinical Epidemiology Program, BLUEPRINT Translational Research Group, Ottawa Hospital Research Institute, Ottawa, ON K1H 8L6 Canada; 5 Regenerative Medicine Program, Ottawa Hospital Research Institute, Ottawa, ON K1H 8L6 Canada; 6 Chronic Disease Program, Kidney Research Centre, Ottawa Hospital Research Institute, Ottawa, ON K1H 8L6 Canada; 7 School of Pharmaceutical Sciences, University of Ottawa, Ottawa, ON K1H 8M5 Canada

**Keywords:** Mesenchymal stromal cells, Extracellular vesicles, Acute lung injury, Acute respiratory distress syndrome, Biodistribution

## Abstract

**Background:**

Mesenchymal stromal cell-derived extracellular vesicles (MSC-EVs) are a promising cell-free therapy for acute lung injury (ALI). To date, no studies have investigated their biodistribution in ALI or discerned the timing of administration for maximal lung targeting, which are crucial considerations for clinical translation. Our study aimed to characterize a mouse model of ALI and establish the distribution kinetics and optimal timing of MSC-EV delivery during lung injury.

**Methods:**

MSC-EVs were isolated by ultracentrifugation alone (U/C) or tangential flow filtration with ultracentrifugation (TFF-U/C) and characterized by nanoparticle tracking analysis and western blot. A lipopolysaccharide (LPS)-induced mouse model of ALI was established to study the inflammatory response over 72 h. ALI was assessed by histological lung injury score, bronchoalveolar lavage fluid cell count and inflammatory cytokines. For biodistribution studies, ALI mice were intravenously administered fluorescently labeled MSC-EVs to determine the optimal timing of administration and organ-specific biodistribution. Live in vivo and ex vivo fluorescence imaging was conducted at various timepoints post-EV injection.

**Results:**

EVs isolated by either ultracentrifugation alone or TFF-U/C displayed comparable size distribution (~ 50–350 nm) and EV marker expression (CD63/81). TFF-U/C generated a 5.4-fold higher particle concentration and 3.9-fold higher total protein when compared to ultracentrifugation alone. From the inflammatory time-course study, cell count and IL-1β peaked in bronchoalveolar lavage fluid at 24 h after ALI induction. MSC-EVs delivered at 24 h (as opposed to 0.5 h, 5 h or 10 h) after disease induction resulted in a 2.7–4.4-fold higher lung uptake of EVs. Biodistribution studies comparing organ-specific MSC-EV uptake showed progressive lung accumulation up to 48 h post-delivery (threefold higher than the spleen/liver), with a decline at 72 h. Importantly, lung EV fluorescence at 48 h in ALI mice was significantly elevated as compared to control mice. The lung tropism of MSC-EVs was further validated as therapeutically inert EVs derived from HEK293T cells accumulated mainly to the spleen and liver with a 5.5-fold lower distribution to the lungs as compared to MSC-EVs.

**Conclusion:**

MSC-EVs exhibit maximal lung accumulation when administered during heightened inflammation at 24 h after ALI induction. This lung tropism suggests that MSC-EVs may serve as a practical rescue treatment for acute inflammatory respiratory conditions.

**Supplementary Information:**

The online version contains supplementary material available at 10.1186/s13287-023-03472-8.

## Introduction

Acute respiratory distress syndrome (ARDS) is a devastating critical illness with high mortality rates (30–50%) [1, 2]. It carries one of the highest costs of any acute care condition due to the need for intensive care management and prolonged hospital stay [3]. During ARDS, localized pulmonary inflammation in response to lung infection or injury leads to vascular hyperpermeability, migration of inflammatory cells into the airways and impaired gas exchange, culminating in respiratory failure. Despite decades of research, there are no curative therapies for ARDS. Preclinical animal studies have highlighted mesenchymal stromal cells (MSCs) as a promising therapy to reduce inflammation, promote tissue regeneration and improve survival in ARDS [4–8]. However, a recent phase IIa clinical trial showed no differences in efficacy between MSCs and placebo control [9]. Post hoc analysis demonstrated a wide range in cell viability from 36 to 85%, thereby highlighting the important technical challenges faced when attempting to manufacture an effective and viable cell therapy.

In the past decade, it has been shown that MSCs do not require tissue engraftment for efficacy [10–13]. Rather, MSCs achieve their protective effects through paracrine mechanisms, including the secretion of nano-sized, membrane-bound particles known as “extracellular vesicles” (EVs) [14–19]. These vesicles carry biologically active cargo that modulate critical cell processes, including programmed cell death, proliferation and inflammation. Hence, MSC-derived extracellular vesicles (MSC-EVs) represent a potential off-the-shelf, cell-free immunomodulatory therapy. In particular, respiratory diseases have become a prominent focus for the development of MSC-EVs [19, 20]. Our recent systematic review found MSC-EVs to be therapeutically effective for diverse lung diseases ranging from acute to chronic and neonatal to adult conditions [21]. Within studies of acute lung injury (ALI, the preclinical correlate of ARDS), a quantitative meta-analysis demonstrated improvements to clinically relevant outcomes, including histological lung injury, lung vascular permeability, inflammatory cell infiltration and mortality.

With rising interest in developing EV therapeutics, many studies have focused on identifying molecular mediators responsible for MSC-EV function. A variety of microRNAs, mRNAs, growth factors and transcription factors encapsulated within MSC-EVs have been discovered to play critical roles in attenuating lung injury [19, 22]. Although these mechanistic insights add to our understanding by which EVs exert their benefits, the National Heart, Lung and Blood Institute (NHLBI) recently highlighted major concerns within the EV field that hinder bench-to-bedside translation [23]. For one, there is still no consensus on the optimal isolation technique to efficiently and consistently produce EVs of high purity. However, the International Society for Extracellular Vesicles (ISEV) guidelines for EV experimentation (“MISEV 2018”) have helped improve rigor in EV isolation and characterization [24]. Moreover, the EV-TRACK international database enables greater standardization in the reporting of study design parameters [25].

Other major knowledge gaps identified by the NHLBI and ISEV are the paucity of evidence comparing different tissue sources, treatment protocols (e.g., timing of administration) and organ uptake of MSC-EVs [23, 26, 27]. We previously reported that less than a quarter of MSC-EV studies conducted biodistribution analyses [19]. Since then, important publications have advanced our understanding of EV pharmacokinetics. One of the first studies in non-human primates found the liver and spleen to be the major site of uptake for EVs generated by a human embryonic kidney-derived cell line (detected up to 24 h after administration) [28]. The development of techniques for genetic modification has also allowed EVs to be conjugated with brain-specific motifs for targeted delivery [29, 30]. However, there is still a lack of biodistribution data within the domain of respiratory diseases. Indeed, our systematic review found only two studies that provided qualitative evidence of EV distribution by fluorescence microscopy of lung sections [31, 32]. Neither study quantitatively assessed the biodistribution of MSC-EVs during ALI, nor did they determine the optimal timing of administration for maximal lung targeting. In other disease states, the distribution pattern of MSC-EVs is linked to regions of injury where pro-inflammatory environments may facilitate vesicle homing [33–35]. Here, we used fluorescently labeled MSC-EVs and in vivo optical imaging techniques to establish the pharmacokinetics and optimal timing of MSC-EV delivery in a preclinical mouse model of ALI (our primary outcome). We hypothesized that during a state of heightened lung vascular permeability and inflammation MSC-EVs would exhibit greater targeting to the lung tissue.

## Materials and methods

### Study approval

Animal study protocols were approved by the Animal Care Committee of the University of Ottawa (protocol no. OHRI-3501) in accordance with the guidelines issued by the Canadian Council on Animal Care. Our manuscript is reported following the ARRIVE guidelines [36].

### Cell culture

Human umbilical cord MSCs (UC-MSCs) were derived from umbilical cords as previously described [37]. Both human bone marrow MSCs (BM-MSCs, Lonza, Basel, Switzerland) and UC-MSCs (University of Dresden) ranging from passage 3 to 6 were cultured in 175-cm^2^ flasks for the production of EVs. BM-MSCs and UC-MSCs were cultured in minimum essential medium α (MEM-α, Thermo Fisher Scientific, MA, US) supplemented with 20% fetal bovine serum (FBS, Thermo Fisher Scientific, MA, US) and penicillin/streptomycin. MSCs were confirmed for their adherence to plastic and tri-lineage differentiation potential. Flow cytometry (Attune Acoustic Focusing cytometer, Invitrogen, CA, USA) was used to detect the expression (CD105, CD73 and CD90) and absence (CD19, CD34, CD14 and HLA-DR) of surface antigens, which were analyzed using the FlowJo 10.0 software (FlowJo, LLC) [38].

HEK293T cells (ATCC, VA, USA) were cultured with Dulbecco’s modified Eagle medium (DMEM, Thermo Fisher Scientific, MA, USA) supplemented with 10% FBS and penicillin/streptomycin in 175-cm^2^ flasks. All cell cultures were maintained in a 37 °C, 5% CO_2_ and 5% O_2_ tissue culture chamber.

### EV isolation and characterization

MSCs and HEK293T cells at 80–90% confluency were cultured for 24 h with serum-free media. The media were collected and centrifuged at 2500 g for 10 min at 4 °C to remove any live cells and apoptotic bodies. The supernatant was collected and frozen at − 80 °C until EV isolation. After media collection, cells were counted using an automated cell counter (Countess™, Thermo Fisher Scientific, MA, USA, or CellDrop™, DeNovix, DE, USA).

Two techniques were employed for EV isolation: (1) *Ultracentrifugation*: Conditioned media were centrifuged at 20,000 g for 20 min. The supernatant was collected and spun at 100,000 g for 90 min at 4 °C (Optima L-100 XP Ultracentrifuge, Rotor 45Ti, Beckman Coulter, CA, USA) and then resuspended into phosphate-buffered saline (PBS, Thermo Fisher Scientific, MA, USA). A second 100,000 g spin (OptimaMAX Ultracentrifuge, Rotor TLA55, Beckman Coulter, CA, USA) was completed before resuspension in PBS for storage at − 80 °C. (2) *Tangential flow filtration with ultracentrifugation (TFF-U/C):* Supernatant was concentrated using a modified polyethersulfone hollow fiber with a 500-kDa filter cartridge (MidGee Hoop Ultrafiltration Cartridge, UFP-500-C-H42LA, GE Healthcare, UK), followed by ultracentrifugation at 100,000 g for 30 min at 4 °C before resuspension in PBS and storage at − 80 °C.

The protein concentration of EVs was determined by DC protein assay (Bio-Rad, ON, Canada). Immunoblot analysis of CD63 and CD81 was used to confirm EV enrichment. Size distribution and particle concentration were determined by ZetaView (Particle Metrix, Meerbusch, Germany).

### Immunoblot

An SDS–polyacrylamide gel electrophoresis of MSC-EVs (5 μg) was performed with 4–20% Mini-PROTEAN® TGX™ Protein Gels (Bio-Rad, ON, Canada). Separated proteins were transferred to nitrocellulose membranes (NOVEX iBLOT Transfer Stacks, Thermo Fisher Scientific, ON, Canada), and membrane blots were blocked with a 5% milk solution in TBS-T (Tris-buffered saline with 0.1% Tween-20). After blocking, blots were incubated overnight at 4 °C with CD63 (1:200, Abcam, Canada) and CD81 (1:200, Santa Cruz, USA), which are primary antibodies specific to the EV proteins. Washed membranes were incubated with an IRDye® 800CW anti-rabbit secondary antibody (LI-COR Biotechnology, NE, USA) before imaging with the Odyssey® imaging system (LI-COR Biotechnology, NE, USA).

### Mouse model of acute lung injury

C57BL/6 male mice (20–25 g), aged 8–12 weeks old (*n* = 126), were used for all animal studies. Mice were purchased from Charles River Laboratories and housed five per cage (conventional cages) with ad libitum access to standard chow and water. Mice were acclimatized for one week at the University of Ottawa animal facility, exposed to a 12/12-h light/dark cycle in a humidity-controlled (30–60% relative humidity) and temperature-controlled (21–24 °C) environment. Three animal trials were conducted. The first trial was a time-course study of inflammatory response and lung injury over 72 h in an LPS-induced mouse model of ALI. The second trial was conducted to determine the optimal timing of MSC-EV administration for maximal lung targeting in LPS-induced ALI mice. The third trial further assessed the biodistribution of MSC-EVs over time (up to 72 h) in healthy and ALI mice. For ALI induction in all trials, mice were anesthetized with ketamine (120 mg/kg)/xylazine (6 mg/kg), orally intubated with a sterile catheter and received 50 μg of intratracheal lipopolysaccharide (LPS, *E. coli* 055:B5; MilliporeSigma, VT, USA) resuspended in 40 μL of PBS. Mice were randomly assigned using a random number generator to one of the following groups: *t* = 1, 3.5, 10, 24 or 72 h post-LPS administration (*n* = 3–5 per group) in the first trial; *t* = 0.5, 5, 10 or 24 h post-LPS administration (*n* = 3 per group) in the second trial; and *t* = 0.5, 3, 10, 24, 48 or 72 h post-MSC-EV administration (*n* = 4–10 per group) in the third trial. For the last trial assessing MSC-EV biodistribution, mice received jugular vein injections of either PBS (background control) or DiR-labeled MSC-EVs. The incision site was sutured after EV infusion, and animal wellness was assessed twice daily.

At the study endpoint in the first trial (inflammatory time-course experiment), animals were anesthetized with 120 mg/kg ketamine and 6 mg/kg xylazine for sample collection while maintaining circulation and spontaneous breathing. The left lung was collected for histological analysis. The right lung was lavaged three times with 500 μL of saline to collect bronchoalveolar lavage fluid (BALF) for outcome analysis. Blood was collected through the inferior vena cava, centrifuged (2000 g at 4 °C, 10 min) for plasma collection and stored at − 80 °C. In trials two and three, animals were under isoflurane anesthesia and exsanguinated by collecting blood through the inferior vena cava. In all three trials, following tissue collection, manual cervical spine dislocation was performed.

### Lung histology

Two separate sections of the left lung were used for histopathological analysis. Lung tissues were fixed in 4% paraformaldehyde, embedded in paraffin and sliced into 5-μm-thick sections with a microtome (Leica Microsystems, Ontario, Canada). Lung sections were adhered to a microscope slide and stained with hematoxylin and eosin. Images were acquired by a Panoramic DESK slide scanner (3DHISTECH, Hungary) and analyzed using CaseViewer (3DHISTECH, Hungary). Five random high-power fields (40 × magnification) per mouse lung were analyzed in a blinded fashion for histological lung injury and scored as per the American Thoracic Society guidelines for experimental ALI [39].

### Optical imaging

The IVIS Spectrum in vivo imaging system (PerkinElmer Inc., Waltham, MA, USA) was utilized to investigate the biodistribution of MSC-EVs and EVs derived from HEK293T cells (HEK-EVs). EVs were labeled with a lipophilic near-infrared dye, 1 1,1′-dioctadecyl-3,3,3′,3′-tetramethylindotricarbocyanine iodide (DiR; Invitrogen, CA, USA), according to manufacturer’s recommendations. Briefly, EVs were incubated with 1 μM of DiR for 15 min, followed by 100,000 g ultracentrifugation at 4 °C before resuspension in PBS. Mice received 15 μg of DiR-labeled EVs via jugular vein injection. Fluorescence imaging was conducted using the IVIS Spectrum system with excitation and emission filters at 745 nm and 800 nm, respectively. To measure the biodistribution of DiR-labeled EVs, regions of interest were gated around ex vivo organs. Fluorescence was measured in units of radiant efficiency, which is the ratio of emission light (photons/sec/cm^2^/sr) to excitation light (μW/cm^2^). In all experiments and at each timepoint, mice treated with PBS generated background fluorescence values that were subtracted from EV-treated organ fluorescence values.

### Measurements of BALF cell count, protein concentration and cytokine analysis

BALF cell concentration was determined using a Countess™ automated cell counter (Thermo Fisher Scientific, MA, USA). BALF was then centrifuged at 800 g (4 °C, 10 min) to pellet cells, while the supernatant was frozen at − 80 °C until further analysis.

BALF protein concentration was measured using a DC protein assay (Bio-Rad, ON, Canada). IL1-β and IL-6 concentrations in BALF and plasma were measured using murine cytokine-specific ELISA kits (Thermo Fisher Scientific, MA, USA).

### Statistical analysis

Data are represented as mean ± SEM unless otherwise stated. All statistical analyses were conducted using GraphPad Prism (GraphPad software, version 9.1.0, San Diego, USA). Statistical analysis was completed using Student’s t-test or one-way ANOVA (> 2 groups) followed by Tukey’s post hoc test for multiple comparisons. Due to the exploratory nature of this work, an a priori sample size was not determined. *P* value < 0.05 was considered significant.

## Results

### Characterization of MSC-EVs by tissue source and isolation method

**Fig. 1:**
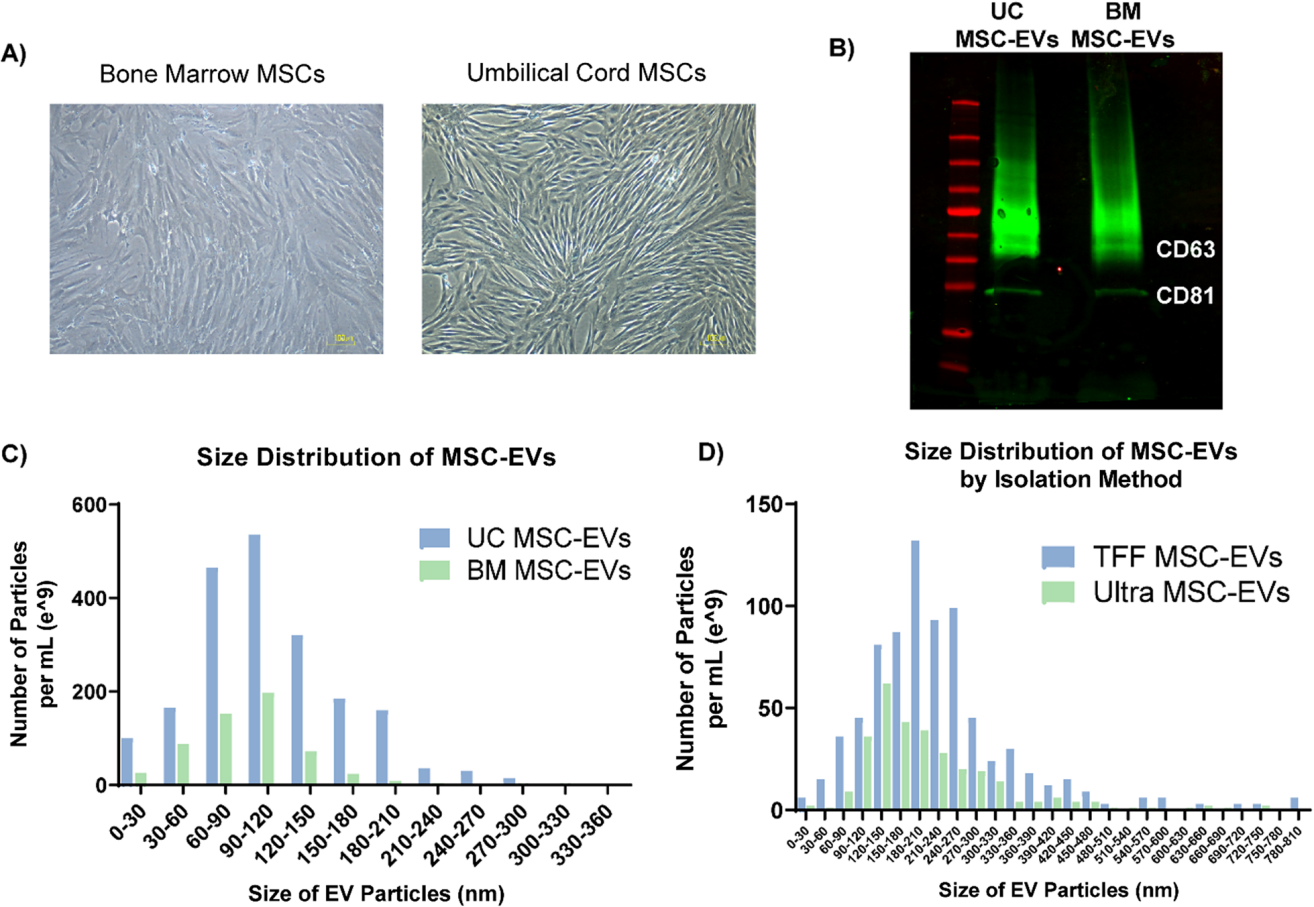
**A** Comparison of cell morphology between bone marrow (BM)- and umbilical cord (UC)-derived MSCs. **B** Western blot analysis of EV protein markers including CD63 and CD81 from both sources of MSC-EVs. **C** Comparison of particle size distribution between UC-MSC-EVs (blue) and BM-MSC-EVs (green). **D** Comparison of particle size distribution between UC-MSC-EVs isolated by tangential flow filtration with ultracentrifugation (TFF, blue) and ultracentrifugation alone (“Ultra”, green)

Bone marrow, the most common source for MSC-EVs, requires an invasive procedure that limits large-scale, efficient MSC isolation [19]. Conversely, umbilical cord tissue has emerged as an attractive, non-invasive source, given its abundance and efficiency in generating MSCs [40, 41]. Hence, we first determined which MSC source may be most effective for EV production. As compared to bone marrow MSCs, UC-MSCs exhibited more typical MSC morphology, including flattened spindle shape enabling increased cell density (Fig. 1A). EVs from both tissue sources displayed CD63/CD81 expression by western blot (Fig. 1B and Additional file 1: Fig. S1). Although umbilical cord-derived EVs exhibited a larger mean size (112.2 ± 52.3 nm, compared to 93.0 ± 39.3 nm for bone marrow), both groups of EVs were within the 30–300 nm size range (median of 89.8 nm for bone marrow and 103.1 nm for UC-MSC-EVs) (Fig. 1C). UC-MSCs produced a threefold higher yield than bone marrow MSCs. Hence, UC-MSCs were selected as the cell type of choice for in vivo biodistribution experiments.

Conditioned media obtained from the same batch of UC-MSC culture were processed by ultracentrifugation alone or TFF-U/C to compare their EV isolation efficiencies. Both methods resulted in EV preparations with comparable size distribution (Fig. 1D). TFF-U/C resulted in 5.4-fold more EV particles and 3.9-fold higher total protein as compared to ultracentrifugation. Given these findings, TFF-U/C was used as the primary isolation technique for subsequent experiments.

### Molecular markers of inflammation peak at 10–24 h after ALI induction in mice

**Fig. 2:**
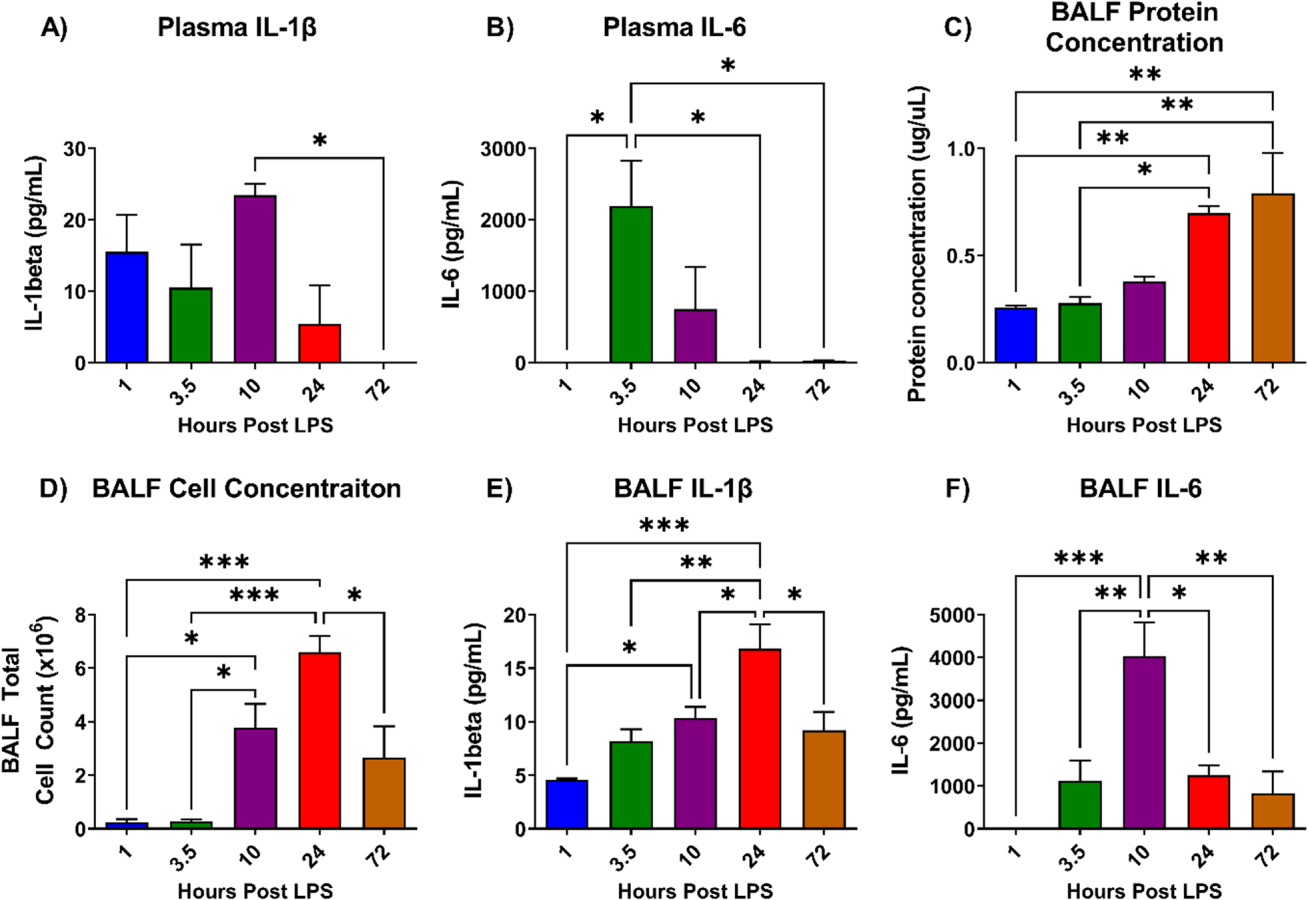
Inflammatory time-course analysis of lipopolysaccharide (LPS)-induced mouse model of ALI. Mice were killed at various timepoints after intratracheal LPS. Plasma concentrations of **A** IL-1β and **B** IL-6 were variable after disease induction. **C** Lung vascular permeability as assessed by bronchoalveolar lavage fluid (BALF) protein concentration peaked at 24 to 72 h. Both BALF **D** cell concentration and **E** IL-1β levels were highest at 24 h after ALI induction, whereas **F** IL-6 levels peaked at 10 h. Data are presented as mean ± SEM, *N* = 3–4 per timepoint, *P < 0.05, **P < 0.01, ***P < 0.001

**Fig. 3:**
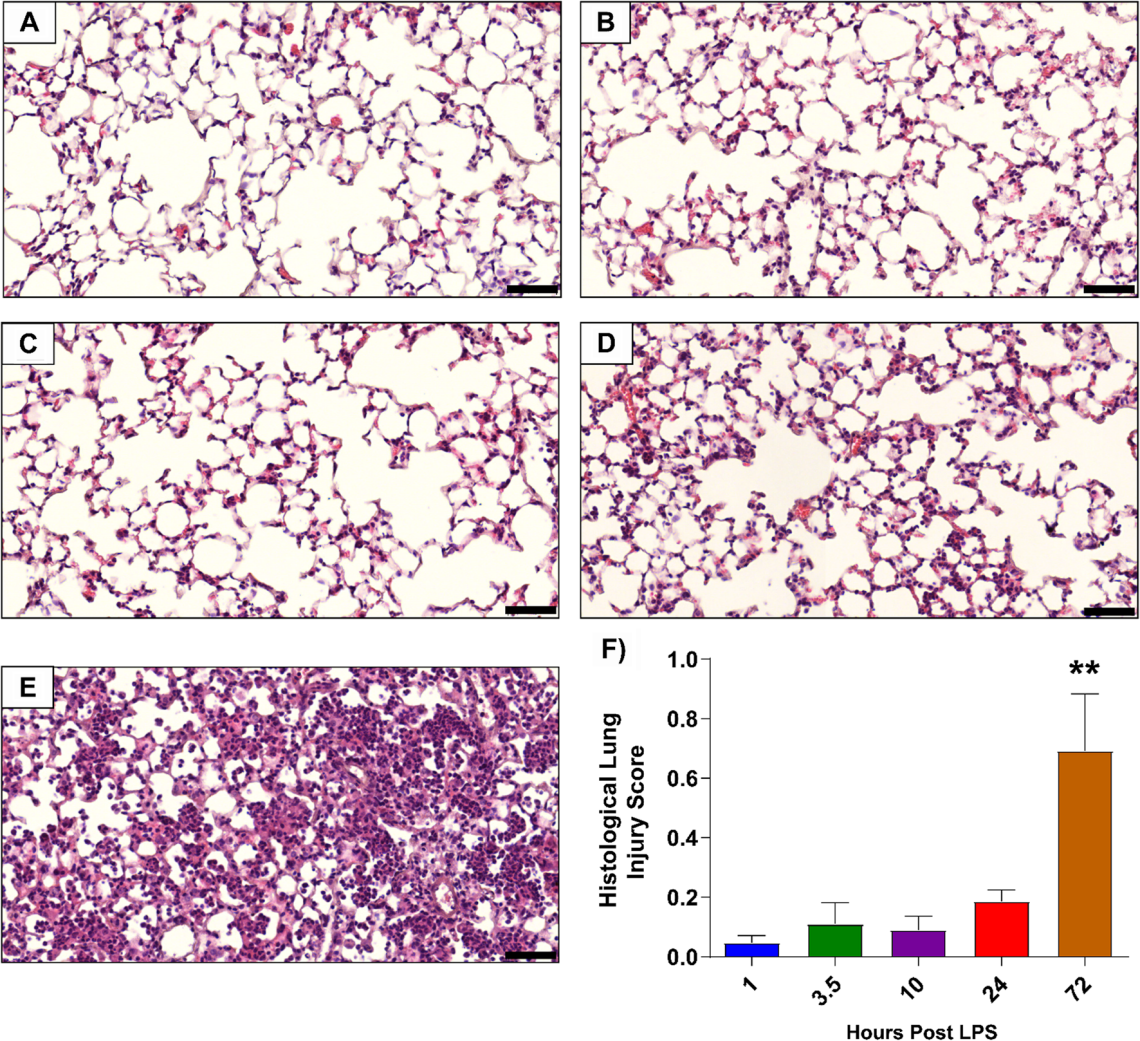
Histological analysis of mice lung sections at various timepoints after LPS-induced ALI. Representative histological images are shown for 1 h, 3.5 h, 10 h, 24 h and 72 h (panels **A**–**E**, respectively). **F** Histological lung injury score was highest at 72 h and evaluated as per the American Thoracic Guidelines, which includes assessment for: (1) intra-alveolar cell infiltration, (2) cell infiltration into interstitial space, (3) hyaline membranes, (4) proteinaceous debris in airspace (e.g., fibrin strands) and (5) alveolar septal thickening [30]. *N* = 3–5 per timepoint. Data are presented as mean ± SEM with a maximum score of 1.0, **P < 0.01. Scale bar represents 50 μm

We conducted a time-course study to determine the inflammatory response in our mouse model of ALI. Following intratracheal administration of LPS, mice were sacrificed at 1 h, 3.5 h, 10 h, 24 h or 72 h. In plasma, variable concentrations in IL-1β and IL-6 were observed with a potential peak in IL-6 at 3.5 h post-LPS (Fig. 2A, B). Lung vascular permeability, as measured by BALF protein concentration, was maximally detected 24–72 h after disease induction (Fig. 2C). BALF cell count and IL-1β levels were highest at 24 h post-LPS, whereas IL-6 levels peaked at 10 h (Fig. 2D, E). These results indicate a localized pulmonary injury from intratracheal LPS administration with a peak in inflammatory response at 10–24 h after disease induction. A delayed response in histopathological lung injury was observed as lung injury score was highest at 72 h after LPS administration (Fig. 3).

### Optimal timing of MSC-EV administration during ALI

**Fig. 4:**
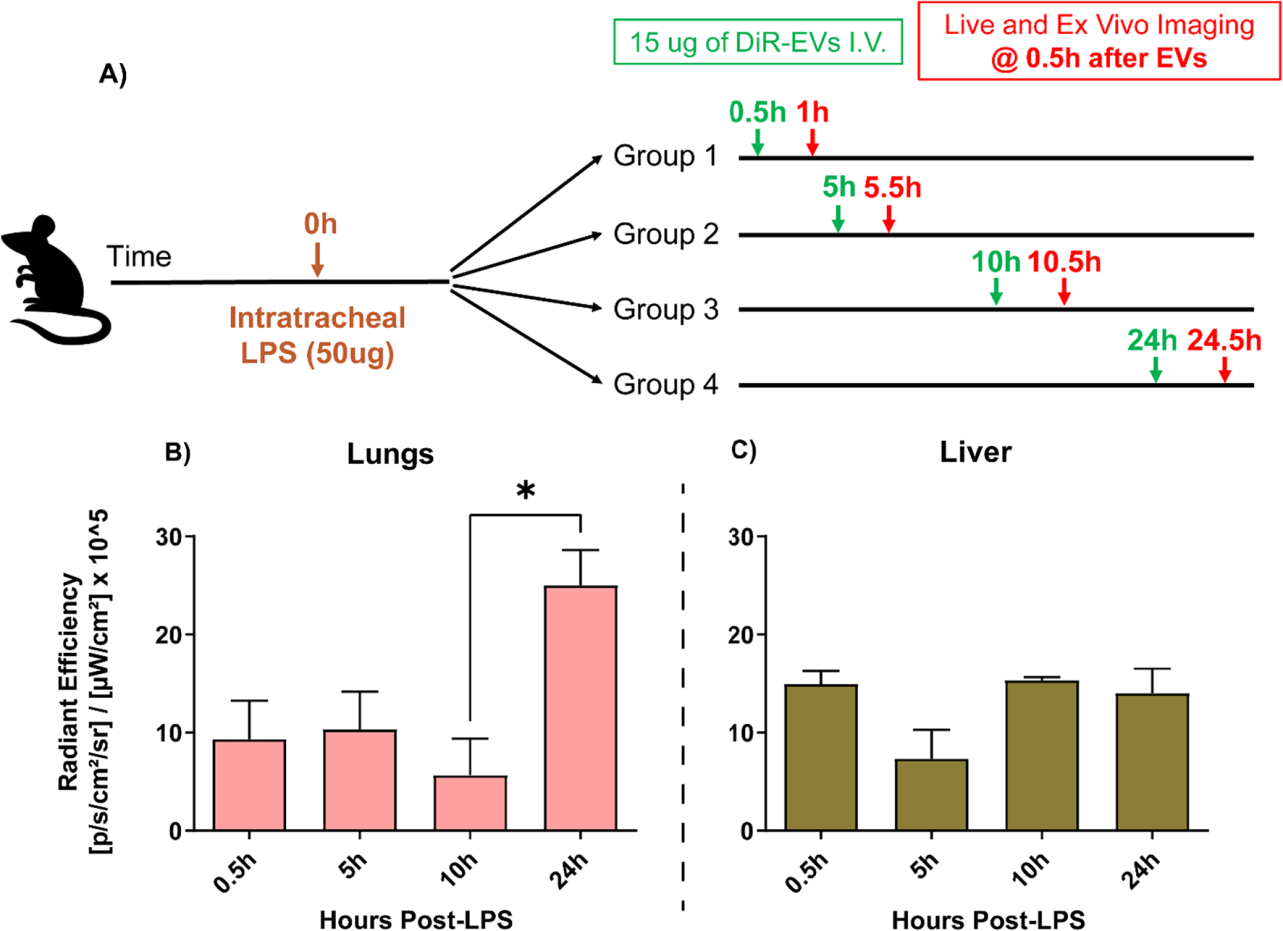
MSC-EVs were labeled with DiR to evaluate the optimal time of administration during ALI. **A** Study design of the experiment including intravenous delivery of DiR MSC-EVs (15 μg) at 0.5 h, 5 h, 10 h or 24 h after disease induction, followed by live and ex vivo fluorescence imaging 0.5 h after EV administration. **B** Lung and **C** liver fluorescence as measured by radiant efficiency. Optimal timing of EV administration for lung accumulation was found to be at 24 h after disease induction. Data are presented as mean ± SEM, *N* = 3 per group, *P < 0.05

DiR-labeled MSC-EVs were intravenously injected into mice at 0.5 h, 5 h, 10 h or 24 h after ALI induction (Fig. 4A). Given the animal skin/tissue barrier and autofluorescence of rodent chow [42, 43], live fluorescence images of mice were assessed qualitatively (Fig. 4B). The fluorescence intensity of ex vivo lungs and livers enabled a quantitative comparison of MSC-EV distribution between groups. EVs injected 24 h post-LPS resulted in enhanced lung accumulation (Fig. 4B), corresponding to a period of heightened lung inflammation and alveolar permeability (Fig. 2). In contrast, the timing of EV administration did not affect the liver distribution of MSC-EVs, as no hepatic inflammatory changes or hyperpermeability was expected (Fig. 4C). Hence, the 24-h administration timepoint was used for subsequent experiments.

### Biodistribution analysis indicates lung tropism by MSC-EVs

**Fig. 5:**
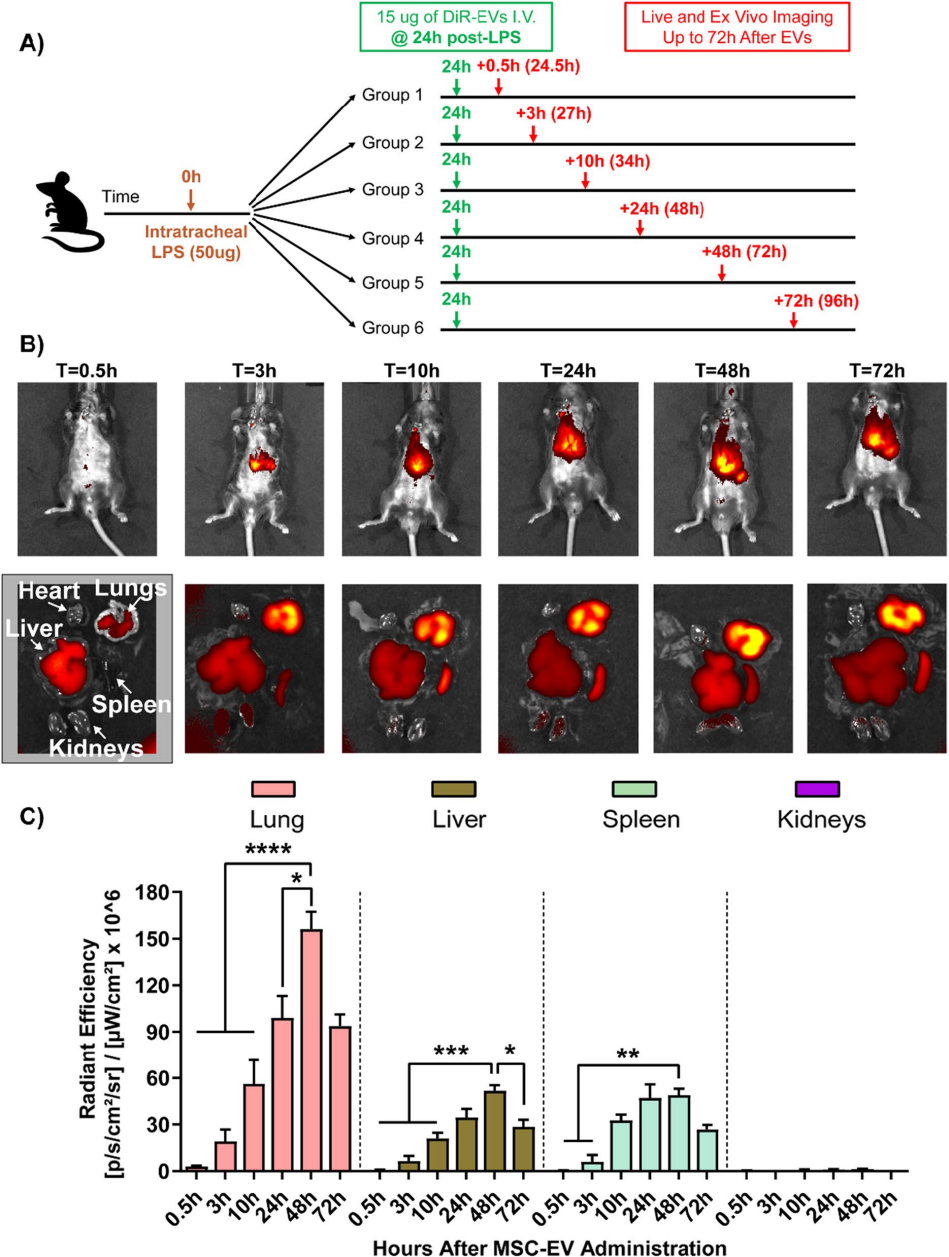
Biodistribution analysis of MSC-EVs during LPS-induced ALI. **A** Study design in which DiR-labeled MSC-EVs were administered at 24 h after ALI induction, followed by fluorescence imaging at various times after EV delivery. **B** Representative live and ex vivo images are shown. **C** Comparison of radiant efficiency between the lungs, liver, spleen and kidneys. Distribution of MSC-EVs was greatest after 48 h, and lung fluorescence was threefold higher than the spleen/liver and 80-fold higher than the kidneys at peak fluorescence. *N* = 4–10 per group. Data are presented as mean ± SEM, *P < 0.05, **P < 0.01, ***P < 0.001, ****P < 0.0001

We next aimed to better understand the kinetics and organ-specific uptake of EVs over time. ALI was induced in mice through intratracheal LPS administration at 0 h, followed by delivery of DiR-labeled MSC-EVs (15 μg) at 24 h. Mice were randomly allocated into groups for live and ex vivo organ imaging at 0.5 h, 3 h, 10 h, 24 h, 48 h or 72 h after EV administration (Fig. 5A). From live mice imaging, greater fluorescence in the lung and liver region was evident over 72 h as EVs were allocated more time to redistribute (Fig. 5B). From quantitative ex vivo organ analysis, we found a progressive increase in lung accumulation of MSC-EVs with a peak at 48 h. Importantly, MSC-EV fluorescence at 48 h in the lungs of mice with ALI was threefold higher than the spleen and liver and 80-fold higher than kidneys (Fig. 5C).

**Fig. 6:**
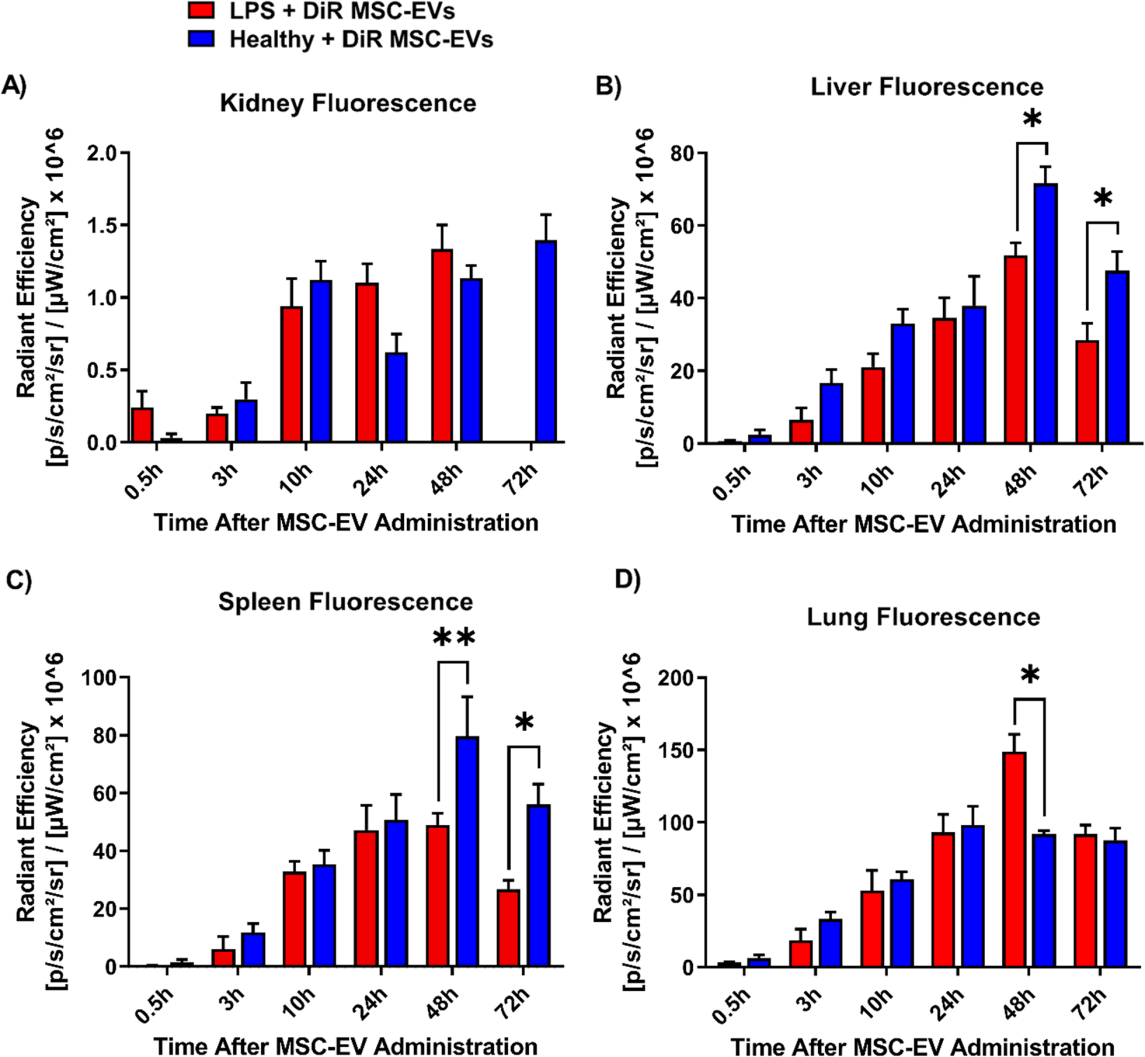
Comparison of DiR-labeled MSC-EV biodistribution between ALI and healthy mice. Ex vivo florescence in **A** kidneys, **B** liver, **C** spleen and **D** lungs found significantly elevated pulmonary distribution during ALI, whereas greater accumulation to the liver and spleen was seen in healthy mice. *N* = 4–10 per group for ALI mice, *N* = 3–6 for healthy mice. Data are presented as mean ± SEM, *P < 0.05, **P < 0.01

Next, we administered DiR-labeled MSC-EVs to healthy mice to determine whether the enhanced lung accumulation of MSC-EVs is only observed during a state of pulmonary inflammation and hyperpermeability. In comparison with ALI, healthy mice demonstrated markedly reduced distribution to the lungs with greater accumulation to the liver and spleen at 48 h (Fig. 6). These findings provide evidence that MSC-EVs exhibit augmented lung tropism during ALI.

### UC-MSCs have markedly elevated EV production as compared to HEK293T cells

HEK293T cells have been widely used in EV research due to their rapid proliferation, high vesicle yield and ease of genetic manipulation [30, 44, 45]. To investigate whether the lung tropism of EVs is specific to MSC-EVs, HEK-EVs were generated. The in vitro efficiency of EV production between UC-MSCs and HEK293T cells was compared (*N* = 4 per group). At confluence in 175 cm^2^, conditioned media were collected from 4.9 ± 0.4 million UC-MSCs, as compared to 12.1 ± 0.2 million HEK293T cells. Utilizing TFF-U/C for EV isolation, UC-MSCs produced 0.46 ± 0.02 μg of EV protein per mL of culture media over 24 h of serum starvation. In comparison, HEK293T cells produced 0.24 ± 0.02 μg of EV protein per mL of media. After normalization to cell number, EV production for UC-MSCs (0.09 μg/million cells) was over fourfold greater than HEK293T cells (0.02 μg/million cells).

### Biodistribution of HEK-EVs indicates high liver and spleen targeting

**Fig. 7:**
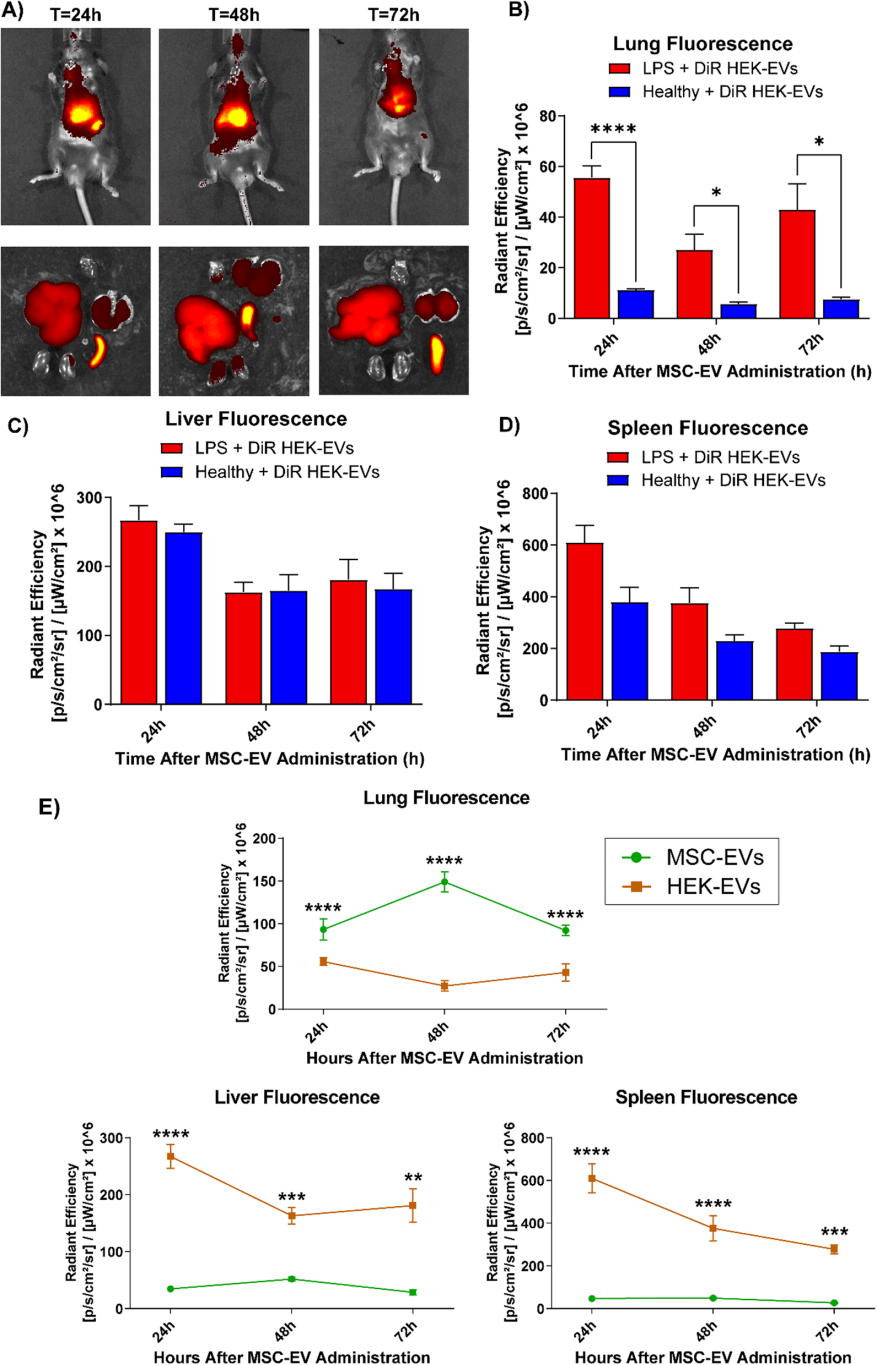
Biodistribution of DiR-labeled EVs derived from HEK293T cells (HEK-EVs). **A** Representative live and ex vivo organ images at 24 h, 48 h and 72 h after HEK-EV administration in ALI mice. Comparison of EV distribution between mice with LPS-induced ALI and healthy controls found significantly elevated **B** lung accumulation of EVs during ALI with no changes to **C** liver or **D** spleen, *N* = 5–6 per group for ALI mice, *N* = 3–6 for healthy mice. **E** Comparison of organ distribution of EVs derived from MSCs and HEK293T cells during ALI, *N* = 4–10 per group for MSC-EVs, *N* = 5–6 per group for HEK-EVs. Data are presented as mean ± SEM, *P < 0.05, **P < 0.01, ***P < 0.001, ****P < 0.0001

To compare the biodistribution pattern of MSC-EVs to HEK-EVs, DiR-labeled HEK-EVs (15 μg) were intravenously injected into mice at 24 h after ALI induction (Fig. 7A). Similar to MSC-EVs, HEK-EVs displayed markedly elevated lung affinity during ALI compared to healthy mice (Fig. 7B). No differences in HEK-EV accumulation to the liver or spleen were observed between ALI or healthy mice (Fig. 7C, D). However, HEK-EVs were found to have a 5.5-fold lower distribution to the lungs as compared to MSC-EVs at 48 h and a 3.1-fold and 7.7-fold greater distribution to the liver and spleen, respectively (Fig. 7E). Hence, MSC-EVs exhibited significantly greater lung tropism as compared to therapeutically inert HEK-EVs.

## Discussion

The field of EV therapeutics is growing at an exponential rate due to their favorable properties as an off-the-shelf, cell-free intervention. Hundreds of articles have provided evidence for the restorative abilities of MSC-EVs in diverse diseases. However, our preclinical systematic review investigating the efficacy of MSC-EVs found a paucity of biodistribution experiments to confirm whether EVs reach their target tissue of interest [19]. Moreover, within the respiratory field, no existing publications have examined the distribution kinetics of MSC-EVs [21]. In this study, we first characterized an LPS-induced mouse model of ALI. Peak inflammatory response and vascular permeability were detected 10–24 h after intratracheal LPS. Accordingly, delivering MSC-EVs at 24 h post-disease induction resulted in enhanced lung accumulation of EVs. To our knowledge, no other studies have investigated the most effective timing of EV administration during ALI. These findings support the possibility of lung localization of MSC-EVs in future clinical trials of ARDS.

It has been reported that the main sites of MSC-EV accumulation are the liver and spleen, with minimal distribution to the lungs [35]. However, our study found that umbilical cord MSC-EVs exhibited high lung tropism that was far greater than any other organ when administered via the jugular vein. These findings were further accentuated during ALI, which can be attributed to increased lung vascular permeability and the potential ‘homing’ effects of MSC-EVs [35]. For example, EVs have limited accumulation in the heart and kidneys of healthy animals [46]. However, in a mouse model of myocardial infarction, deposition of MSC-EVs in the infarcted heart was markedly elevated as compared to healthy animals due to increased coronary vascular permeability [47]. Similarly, during acute renal injury, preferential targeting of MSC-EVs to the damaged kidneys can also be observed [33]. Proteomic profiling of macrophage-derived EVs revealed integrin expression as a large proportion of small EV membrane proteins [48]. These cell adhesion molecules were critical in the selective binding of EVs to the kidneys during ischemic–reperfusion injury, which was otherwise negligible in healthy animals. The authors further found that EV accumulation gradually increased with prolonged ischemia [48]. Similarly, in our study, the peak in EV distribution corresponded to maximal histological lung injury at 48 h after EV delivery (72 h after disease induction). Furthermore, MSCs are known to express chemokine receptors on their cell surface for homing to sites of inflammation [49]. In particular, overexpression of CXCR4 in MSCs augmented cellular migration, in vitro paracrine function and in vivo immunomodulation in a rat model of ALI [50]. Hence, chemokine receptors like CXCR4 and others on MSC-EVs may also be facilitating their ability to preferentially target injured tissues in various diseases. This will need to be explored in future studies.

Interestingly, we observed that lung tropism was specific to MSC-EVs, whereas HEK-EVs mainly accumulated in the liver and spleen. A recent systematic review of EV biodistribution data illustrates that this disposition pattern of HEK-EVs is consistent with what is commonly observed in EVs from several cell sources [46]. HEK-EVs, unlike MSC-EVs, lack innate therapeutic effects and their deposition into the liver and spleen leads to rapid clearance. In contrast, EVs derived from cancer cells are known to exhibit a strong affinity for the lungs as a common site for metastases [46]. This pattern of distribution is mediated by two important surface membrane proteins, integrins α6β4 and α6β1 [51, 52]. Potential expression of these integrins on MSC-EVs suggests a possible molecular explanation for their biodistribution profile. Nonetheless, MSC-EVs hold promise as a practical intervention for delivering their immunomodulatory effects to the lungs. Future studies could enhance the expression of specific integrins to explore the development of lung-targeted EV therapeutics.

There are limitations in our study that warrant discussion. Although we did not conduct an interventional in vivo study, the therapeutic efficacy of MSC-EVs for ALI has been well documented and our recent meta-analysis of these studies provides further evidence for their protective properties [21]. Due to the paucity of biodistribution data, we decided to focus our efforts on addressing the important knowledge gaps of EV distribution and timing of administration during respiratory injury. Of note, we used DiR, a lipophilic membrane dye, for our imaging studies. The fusion of bioluminescent tags to EV proteins (e.g., CD63) has been shown to alter the physiological biodistribution of EVs, which may produce findings that is not representative of unaltered MSC-EV pharmacokinetics [53]. While there may be alternative methods that offer increased specificity, including membrane radiolabeling or engineering EVs with bioluminescent tags, DiR offers high sensitivity during ex vivo organ imaging, efficient vesicle labeling and no requirements for molecular modifications [53].

There is still no consensus on the gold standard technique for EV isolation, which adds to the heterogeneity in EV products being manufactured and trialed today. Our study observed a more efficient production of EVs when utilizing TFF-U/C combined with umbilical cord MSCs as opposed to ultracentrifugation and bone marrow tissue. Ultracentrifugation is the most frequently applied technique for EV enrichment due to its widespread availability and isolation specificity [19]. However, potential aggregation of proteins, limitations on the amount of material that can be processed and its inefficient yield of EVs are considerable limitations to clinical scale-up [54, 55]. More recently, TFF has become an appealing approach with its minimal restrictions on media volume and absence of centrifugal force that prevents vesicle damage [19]. Previous articles have similarly demonstrated markedly improved EV yield, purity and batch-to-batch consistency, in addition to potentially greater efficacy from TFF isolation [56–58]. Moving forward, more direct head-to-head in vivo comparisons of EVs derived from various cell sources and isolation methods are needed to discern the most effective therapeutic product for clinical translation.

## Conclusion

In summary, we found that intravenous delivery of MSC-EVs 24 h after the onset of respiratory injury resulted in maximal lung accumulation. Notably, MSC-EVs exhibited enhanced pulmonary tropism, evident by the lungs being their major organ distribution site, followed by the liver and spleen, with negligible accumulation to the kidneys. This lung tropic effect of MSC-EVs may reflect their inflammatory homing; in contrast, HEK-EVs were mainly distributed to the liver and spleen. Overall, the preferential uptake of MSC-EVs by the lungs during ALI highlights their potential as a promising rescue therapy for acute inflammatory respiratory diseases.

### Supplementary Information

**Additional file 1: Fig. S1.** Full-length immunoblot image. The first lane represents the protein ladder imaged in red fluorescence. The second lane represents umbilical cord-derived MSC-EVs imaged in green fluorescence. The third lane was empty. The fourth lane represents bone marrow-derived MSC-EVs imaged in green fluorescence. All other lanes were empty and were not included in the region of interest for fluorescence imaging of the immunoblot.

## Data Availability

The dataset supporting the conclusions of this article is available in the Mendeley Data repository, https://data.mendeley.com/datasets/6fpk9ytssj/draft?a=56d780a3-bb31-4849-b444-e558da634b28.

## Abbreviations

ALI: Acute lung injury
ARDS: Acute respiratory distress syndrome
BALF: Bronchoalveolar lavage fluid
BM-MSC: Bone marrow-derived mesenchymal stromal cell
DiR: DiIC18(7); 1,1′-dioctadecyl-3,3,3′,3′-tetramethylindotricarbocyanine iodide
EV-TRACK: Transparent reporting and centralizing knowledge in EV research
HEK-EV: Human embryonic kidney cell-derived extracellular vesicles
ISEV: International society for extracellular vesicles
MISEV: Minimal information for studies of EVs
MSC-EV: Mesenchymal stromal cell-derived extracellular vesicle
TFF: Tangential flow filtration
U/C: Ultracentrifugation
UC-MSC: Umbilical cord-derived mesenchymal stromal cell
