# Supplementary material for: Biodistribution of mesenchymal stromal cell-derived extracellular vesicles administered during acute lung injury
Source: Stem Cell Res Ther. 2023 Sep 13;14:250. doi: 10.1186/s13287-023-03472-8 (PMC10500845; doi:10.1186/s13287-023-03472-8)
Supplement: Supplementary file 1 — Additional file 1: Fig. S1. Full-length immunoblot image. The first lane represents the protein ladder imaged in red fluorescence. The second lane represents umbilical cord-derived MSC-EVs imaged in green fluorescence. The third lane was empty. The fourth lane represents bone marrow-derived MSC-EVs imaged in green fluorescence. All other lanes were empty and were not included in the region of interest for fluorescence imaging of the immunoblot. [file 13287_2023_3472_MOESM1_ESM.docx]

**Additional file 1**


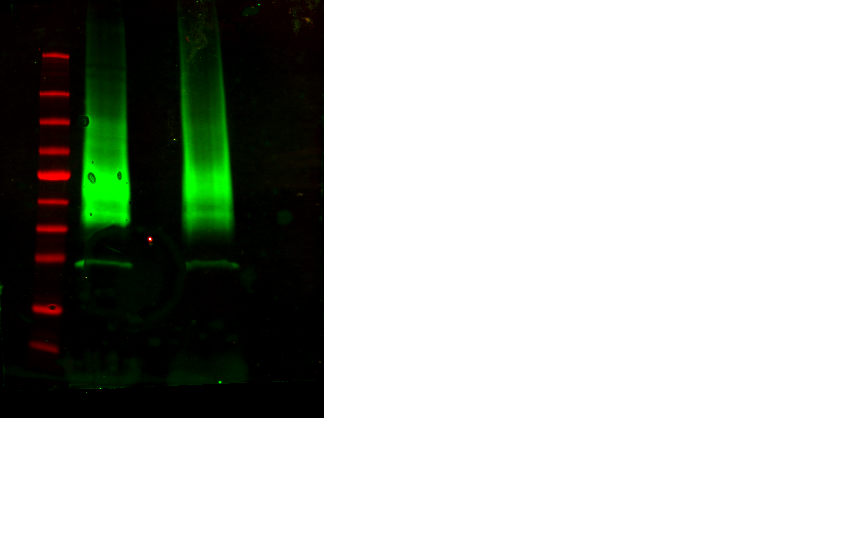


**Figure S1**. Full length immunoblot image. First lane represents the protein ladder imaged in red fluorescence. Second lane represents umbilical cord-derived MSC-EVs imaged in green fluorescence. Third lane was empty. Forth lane represents bone marrow derived MSC-EVs imaged in green fluorescence. All other lanes were empty and were not included in the region of interest for fluorescence imaging of the immunoblot.
